# Adaptive Weibull Multiplicative Model and Multilayer Perceptron Neural Networks for Dark-Spot Detection from SAR Imagery

**DOI:** 10.3390/s141222798

**Published:** 2014-12-02

**Authors:** Alireza Taravat, Natascha Oppelt

**Affiliations:** Remote Sensing & Environmental Modelling Lab, Kiel University, Kiel 24098, Germany; E-Mail: oppelt@geographie.uni-kiel.de

**Keywords:** segmentation, neural networks, dark spot detection, Synthetic Aperture Radar (SAR)

## Abstract

Oil spills represent a major threat to ocean ecosystems and their environmental status. Previous studies have shown that Synthetic Aperture Radar (SAR), as its recording is independent of clouds and weather, can be effectively used for the detection and classification of oil spills. Dark formation detection is the first and critical stage in oil-spill detection procedures. In this paper, a novel approach for automated dark-spot detection in SAR imagery is presented. A new approach from the combination of adaptive Weibull Multiplicative Model (WMM) and MultiLayer Perceptron (MLP) neural networks is proposed to differentiate between dark spots and the background. The results have been compared with the results of a model combining non-adaptive WMM and pulse coupled neural networks. The presented approach overcomes the non-adaptive WMM filter setting parameters by developing an adaptive WMM model which is a step ahead towards a full automatic dark spot detection. The proposed approach was tested on 60 ENVISAT and ERS2 images which contained dark spots. For the overall dataset, an average accuracy of 94.65% was obtained. Our experimental results demonstrate that the proposed approach is very robust and effective where the non-adaptive WMM & pulse coupled neural network (PCNN) model generates poor accuracies.

## 1. Introduction

Remote sensing is a critical element for an effective response to marine oil spills and it is useful in several modes of oil spill control and detection, including large area surveillance, site specific monitoring and tactical assistance in emergencies [1]. Among all remote sensing techniques, the ability of Synthetic Aperture Radar (SAR) imaging to detect features on the ocean's surface makes this technology a powerful tool for monitoring oil spills [2–4]. Since SAR is an active sensor using microwave bands (l-Band (1–2 GHz), C-Band (4–8 GHz), X-Band (8–12 GHz)), it has day/night imaging capability and the ability to penetrate cloud cover [5–7].

On the ocean, the main backscattering process is surface scattering. The elementary scatterers are the waves whose wavelength satisfies the Bragg resonance condition, LBragg = λ/(2 sin θi), where LBragg is the wavelength of small-scale waves, λ is the radar wavelength and θi is the incidence angle [8].

The ocean surface behaves as a mirror without Bragg waves, and most of the incident signal is reflected away in the specular direction. Oil spills damp small-scale Bragg waves and reduce the friction velocity more than oil-free surface because of their larger surface tension than water [8]; in a SAR image they therefore appear as regions with less brightness.

High frequency bands (X & C band) are mainly used for oil spill detection because the damping effect is larger for the Bragg waves of shorter wavelengths. However, the use of l-band for extracting the dark features has already been demonstrated using ALOS-PALSAR and UAVSAR sensors [8–10].

Another factor influencing the backscattering behavior of the ocean surface is the wind speed. The wind speeds under which the dark features of oil spill can be distinguished from surrounding waters are approximately 3–14 m/s [11]. Wind speeds of less than ∼3 m/s smoothens equally the surfaces with and without oil spill; wind speeds higher than ∼14 m/s churn the sea and roughen the surface resulting in a dumping effect which becomes negligible [8].

Oil dispersion also affects the visibility of oil spill in SAR images which is caused by dissolution, oxidization, and biodegradation. Thus, with increasing time from the oil discharge and with increasing wind speed, oil slicks become undetectable [8].

In general, oil spill detection is performed in three steps: dark spot detection (both oil spills and look-alikes), feature extraction and classification (necessary for discriminating oil spills from look-alikes which are ice, internal waves, natural organics, algae, and rain cells) [6,7,12–15]. The literature describes many efforts to develop automated and semi-automated systems for oil spill detection based on this procedure [16,17]. This study focuses on accurate dark-spot detection which is the most computationally intensive phase of the algorithm and is a critical step prior to feature information extraction and classification [18,19].

A variety of algorithms for oil spill segmentation have been developed using different methodologies such as thresholding [15,20], support vector machines [21], and neural networks [22,23]. The approach presented in this paper is a combination of adaptive Weibull Multiplicative Model (WMM) and MultiLayer Perceptron (MLP) neural networks. The results will be compared with the results of Taravat, *et al.*, which is a model from the combination of WMM and pulse coupled neural network (PCNN) techniques to differentiate between dark spots and background signals [3]. The reason for comparing the presented results with the non-adaptive WMM & PCNN results is to test the capability of the adaptive WMM & MLP model for increasing the accuracy of full automatic dark spot detection by using the non-adaptive WMM & PCNN model.

In pulsed coupled neural networks model the setting of parameters represents the fundamental but complex task. Although it has already been proofed that pulsed coupled neural networks is very fast and accurate in dark spot detection, it still generates poor accuracies in some cases (not well-defined linear dark spots and not well-defined massive dark spots) [3].

The presented approach overcomes the non-adaptive WMM filter setting parameters by developing an adaptive WMM model. Furthermore a pixel based MLP neural network has been applied to check the capability of a pixel based classification in order to increase the accuracy of the model discussed in Taravat *et al.* [3].

## 2. Methods

The first step of dark feature detection is applying a filter [11,20]. Liu *et al.* used a 3 × 3 Lee filter, followed by a 5 × 5 Lee filter and a 7 × 7 Median filter applied to the original image [24]. Topouzelis *et al.* used a combination of the Lee and Local Region filters [22]. The combination applied to his study includes application of a 3 × 3 Lee filter to the original image, followed by a 5 × 5 Lee filter and a 7 × 7 Local Region filter [13]. In all of these kind of filters, it has been assumed that the real and the imaginary parts of the received wave follow Gaussian distribution which is in turn lead to Rayleigh distribution [25]. Another popular filter is the Weibull Multiplicative Filter which has shown high degree of success in modeling sea clutter [3,26].

### 2.1. Adaptive Weibull Multiplicative Filter (WMM)

In this study WMM (with the assumption that the amplitude or the intensity image has the Weibull distribution [3]) is used in order to remove speckle and to enhance the contrast between the dark spot and the background [3,26]. The extraction of the texture image from the Weibull-distributed SAR image employs the local estimation of the scale and form parameters of the Weibull distribution [3,26];

The Weibull-distributed random variable *x* with form parameter *γ_x_* > 0 and scale parameter *β_x_* > 0, has a probability density function given by:

(1) $$f\left(x\right)=\frac{\gamma_{x}}{\beta_{x}}\;{\left(\frac{x}{\beta_{x}}\right)}^{\gamma_{x}-1}\text{exp}\;\left[-{\left(\frac{x}{\beta_{x}}\right)}^{\gamma_{x}}\right]$$

The m-order moment can be expressed as:

(2) $$E\left[x^{m}\right]=m\beta_{x}^{m}\;\Gamma (\text{m}/\gamma_{x})/\;\gamma_{x}$$

For *γ_x_* = 2, the Weibull distribution becomes a Rayleigh distribution, for *γ_x_* = 1, it becomes an exponential distribution. It can be shown that *x^a^* with *a* > 0 is also Weibull distributed. If *z* = *x^a^* (*z*) with form and scale parameters given by, *γ_z_* = *γ_x_*/*a* and $\beta_{z}=\beta_{x}^{a}$ follows that:

(3) $$f\left(z\right)=\frac{\gamma_{z}}{\beta_{z}}\;{\left(\frac{z}{\beta_{z}}\right)}^{\gamma_{z}-1}\text{exp}\;\left[-{\left(\frac{z}{\beta_{z}}\right)}^{\gamma_{z}}\right]$$

Consider *b*, with *a* > *b* > 0 in such a way that:

(4) $$\text{z}=\;x^{a}=\;x^{b}x^{a-b}=\frac{x^{b}}{E\left[x^{b}\right]}\;E\left[x^{b}\right]x^{a-b}=st$$

where *s* is the speckle, with unitary mean and *t* is the texture of the Weibull-distributed variable *z*. *z* is the variable for the SAR image.

(5) $$\text{s}=\;x^{b}/E\left[x^{b}\right]\;\text{t}=\;x^{a-b}E\left[x^{b}\right]$$

In this form, it is possible to express *z* as a multiplication of *s* by *t*, where s is the speckle and *t* is the texture of the Weibull-distributed variable *z*. The texture t has Weibull distribution with form and scale parameter given, respectively, by:

(6) $$\gamma_{t}=\overset{\gamma_{x}}{\;}/\underset{\left(a-b\right)}{\;}\;\beta_{t}=\beta_{x}^{a-b}E[x^{b}]$$

and the speckle has Weibull distribution with form and scale parameter given, respectively, by:

(7) $$\gamma_{s}=\overset{\gamma_{s}}{\;}/\underset{b}{\;}\;\beta_{s}=\beta_{x}^{b}/E[x^{b}]$$

**Figure 1.**
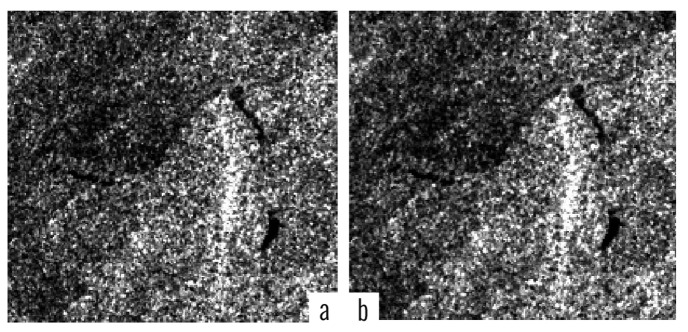

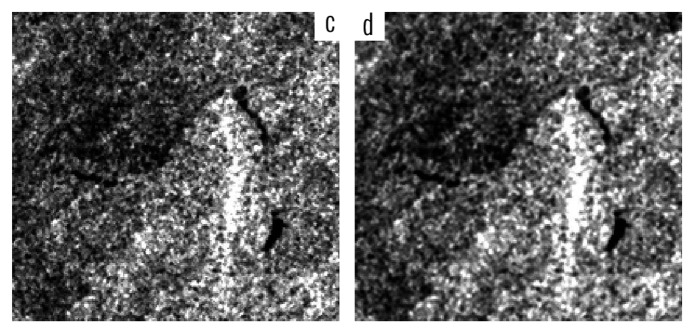
Shows the effect of P parameter in an amplitude SAR C-band ENVISAT image. (**a**) The original image; (**b**) *P* = 0.2, window 3 × 3; (**c**) *P* = 0.5, window 3 × 3; (**d**) *P* = 0.8, window 3 × 3.

Let *p* = *b*/*a*,0 ≤ *p* < 1 Then

(8) $$\text{t}=\;x^{a-b}E\left[x^{b}\right]=\;x^{a(p-1)}E\left[x^{ap}\right]=\;z^{\left(1-p\right)}E\left[z^{p}\right]$$

using p-order moment equation E[*z^p^*]:

(9) $$t=p\beta_{z}^{p}\Gamma (\text{p}/\gamma_{z}){\text{z}}^{1-p}/\;\gamma_{z}$$

where *t* can be considered as the filtered image and the factor 0 ≤ *p* < 1 (which is set manually in non-adaptive WMM) gives the filtering intensity (Figure 1). In adaptive WMM model, the form parameter *γ_s_* can be set as the mean or mode of *γ_z_* in the whole image. Using $\gamma_{s}=\overset{\gamma_{x}}{\;}/\underset{b}{\;}$, it can be obtained that *γ_x_* = *γ_s_b*. Through *γ_z_* = *γ_x_*/*a* and *p* = *b*/*a* < 1, *p* can be calculated adaptively as a function of *γ_z_* that is estimated locally as *γ_z_*/*γ_s_* and the texture becomes:

(10) $$t=\beta_{z}^{\gamma_{z}/\gamma_{s}}\;\Gamma (1/\gamma_{s}){\text{z}}^{1-(\gamma_{z}/\gamma_{s})}/\;\gamma_{s}$$

If *γ_z_* → *γ_s_* there is a stark filtering in the image and if *γ_z_* ≪ *γ_s_* there is a weak filtering and, if *γ_z_* > *γ_s_* the texture equation holds, but it is not Weibull-distributed anymore.

### 2.2. MultiLayer Perceptron (MLP) Neural Networks

The next step is segmenting the filtered image, in which the pixels are grouped according to the similarities by using MLP neural networks. A neuron k can be described by writing the following pair of equations [27]:

(11) $$\begin{array}{c} u_{k}=\overset{n}{\underset{i=1}{\text{∑}}}w_{ki}x_{i}\\ y_{k}=\varphi (u_{k}+b_{k})\;{\left(x+a\right)}^{n}=\sum_{k=0}^{n}\left(\begin{array}{c} n\\ k \end{array}\right)x^{k}a^{n-k} \end{array}$$

where *x*_1_, … … … …, *x_n_* are the input signals, *w_k_*_1_, … … … …, *w_xn_* are the synaptic weights of neuron k, *u_k_* is the linear combiner output due to the input signals, *b_k_* is the bias, *φ*(.) is the activation function, and *y_k_* is the output signal of the neuron. The logistic function is an example of a sigmoidal function which is the most commonly used activation function [1] is defined by the following equation where *a* > 0 is the slope parameter:

(12) $$\varphi \left(v\right)=\;\frac{1}{1+e^{-av}}$$

The logistic function ranges from 0 to +1; however it is desirable to have the activation function range from −1 to +1 (anti-symmetric form). For the corresponding form of a sigmoid function we may use the hyperbolic tangent function, defined by:

(13) c(v)=tanh(v)

Allowing an activation function of the sigmoid type to assume negative values as prescribed by the above equation has analytic benefits [27].

Among neural network architectures, multilayer feedforward networks with MLPs as the learning algorithm are extensively used in pixel based oil spill segmentation [21,22]. Typically, MLPs consists of the input layer, one or more hidden layers of computation nodes, and an output layer of computation nodes. The input signal propagates through the network in a forward direction, on a layer-by-layer basis.

Normalization is a preliminary phase of MLP segmentation which ensures that the distance measures respond with equal weight for each input [22]. Normalization performs by linear transformation from the image interval [0, 255] to the neural networks interval [−1, 1] [22].

IDL programming language and the Stuttgart Neural Network Simulator (SNNS) developed at the University of Stuttgart, Germany [28], has been used for developing the WMM model and the classification algorithm implementation. The WMM filter model be obtained in IDL from website [29].

## 3. Results and Discussion

The model has been tested on a dataset of ENVISAT-ASAR (ASAR Image Mode Medium-resolution Image (IMM) and Wide Swath Mode (WSM) products which have a spatial resolution of 150 m) and ERS2-SAR (PRecision Image (PRI) product which has a pixel size of 12.5 m × 12.5 m) images.

The dataset has been categorized into four groups which are massive well-defined, linear well-defined, massive not well-defined, linear not well-defined, (See [3]: Table 1) based on different types of dark spot and different sea status.

Radiometric calibration and geometric correction have been applied to the dataset in order to generate a backscatter (σ^0^) image, and to georeference the input images into the UTM projection with the WGS84 as datum [3]. After calibration process, sub images containing all potential anomalies detected under a variety of sea conditions were extracted to make extraction more expedient. The test dataset contains 40 images with 256 × 256 pixels (around 4 km^2^), 20 images with 512 × 512 pixels (around 36 km^2^).

**Figure 2.**
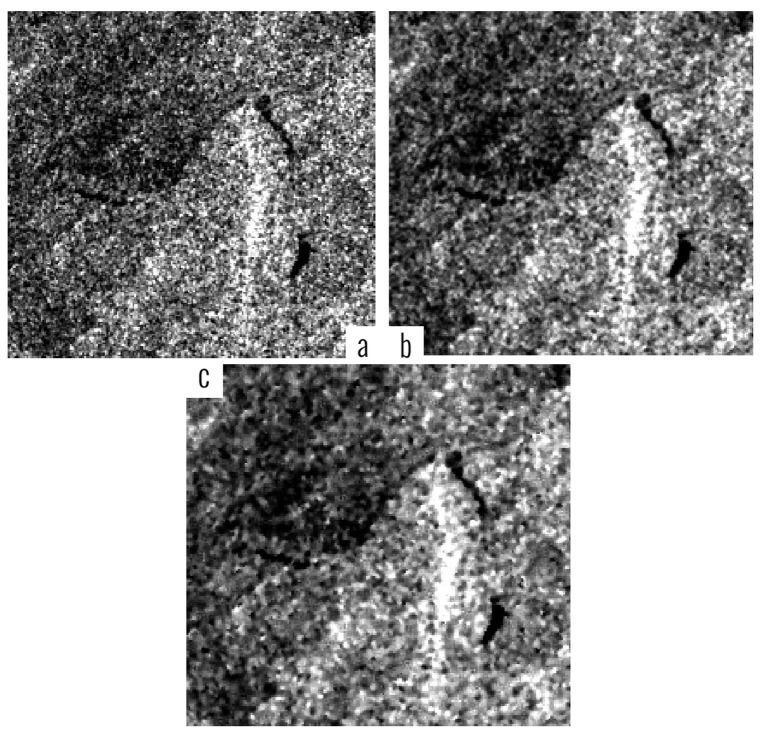
Shows an example of adaptive (**b**) and non-adaptive (**c**) WMM filtering of the original image (**a**).

We applied adaptive WMM filter to all 60 test images. The similarity of adaptive and non-adaptive WMM filtered image has been shown in Figure 2. In the example shown in Figure 2, the filtering intensity P = 0.7 and a 3 × 3 window has been used for non-adaptive WMM (which was found to be the most appropriate combination of parameters for the WMN filter based on the previous studies) [3].

Removing the noisy pixels in the images by using adaptive WMM overcomes the non-adaptive WMM filter setting parameters while preserving the same accuracy of the non-adaptive model.

The classification task is based on the MLP approach. In designing the MLP model the number of units in the hidden layer and the training/testing phase settings (number of training cycles and the pixel selection for training/test the model) represent the fundamental tasks. Adjustment of these parameters affects the capability and sensitivity of the model to fit at the dynamic range of the backscattering values in the scene.

Several attempts have been made to properly select the number of units to be considered in the hidden layers. For training/test of the neural net 7000 pixels were extracted from different types of dark spots and different sea status. The tested windows were chosen to be as different as possible in order to test the neural networks ability to generalize different types of dark formations. The training and test sets were independent, the former containing 60% and the latter 40% of all pixels.

**Figure 3.**
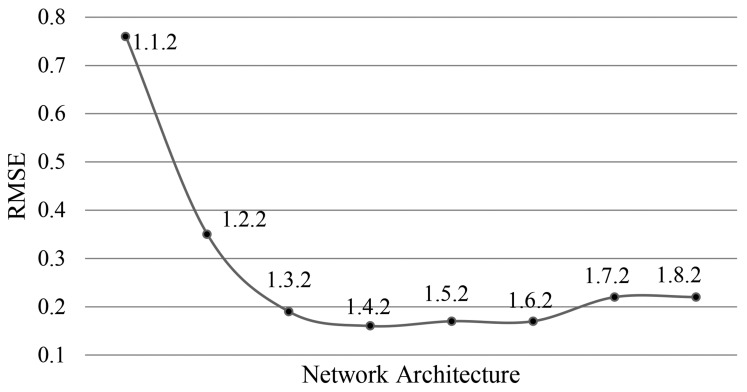
RMSE errors for different neural network topologies.

Pixel selection for training/test set has been done randomly and repeated six times. The presented results of root mean square error (RMSE) errors in Figure 3 are the average of these repetitions for each topology. The topology 1-4-2 has been finally chosen for its good performance in terms of classification accuracy, RMSE, and training time. A number of about 5000 training cycles was sufficient to get the network trained. The input of the net is the filtered image and the output providing classified pixels in terms of oil spill or others. However, after training the network for one specific sensor and product, no further tuning is necessary. After the segmentation phase, a very simple filtering process is used to eliminate all the objects with an area of less than 20 pixels from the processed image [3].

One MLP neural network (with the topology 1-4-2) has been used for classifying all images. In the next phase, for accuracy assessment, 500 pixels have randomly been selected from each sub-image and then labeled (oil spill or others) by visual interpretation. The results have been compared with the results of non-adaptive WMM & PCNN model which is the most recent method in literature presented by Taravat *et al.* [3].

In some cases of the Wide Swath products non-adaptive WMM & PCNN generates poor accuracies because the strong variation of incidence angle from near to far range affects the dynamic range in the images [3].

**Figure 4.**
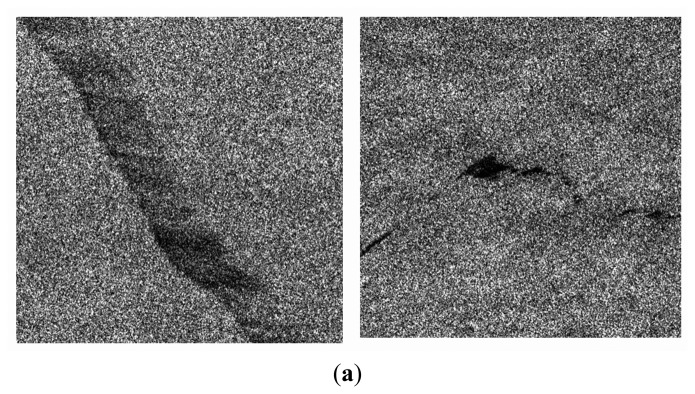

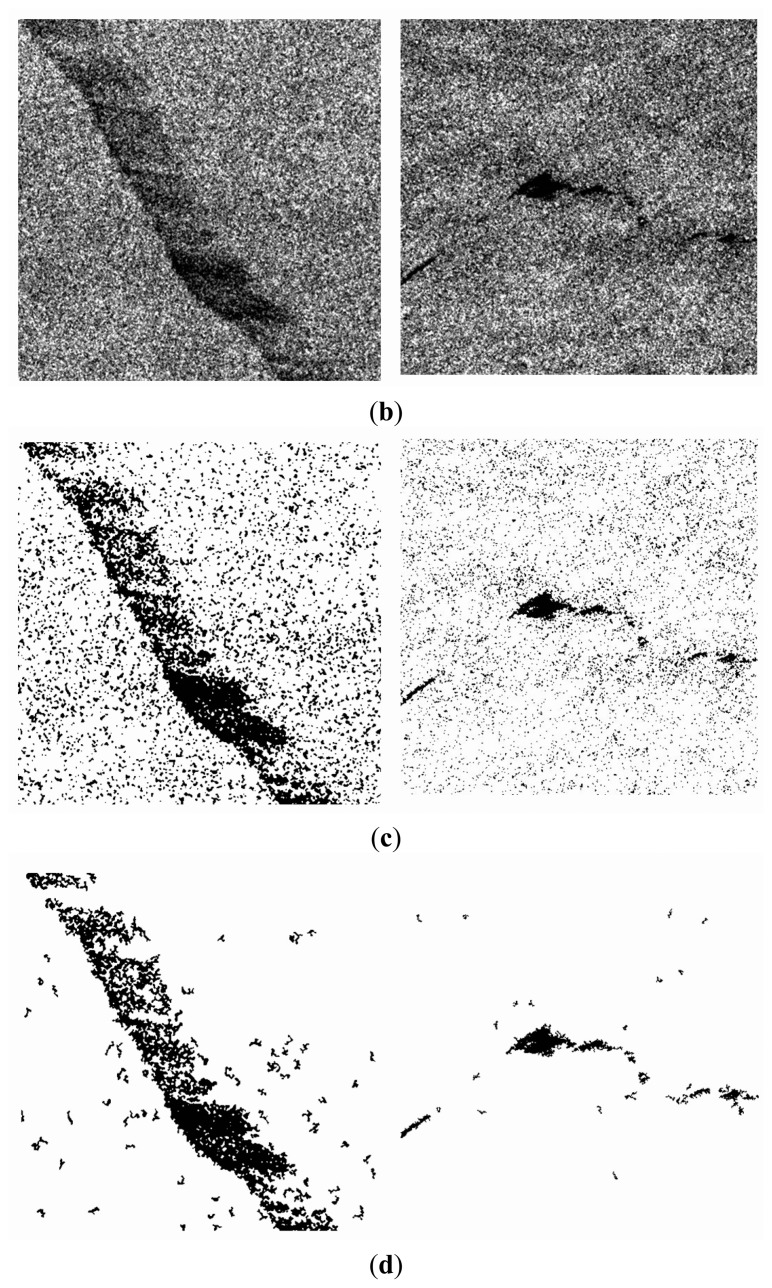
Results of the proposed approach on two typical examples where non-adaptive WMM & PCNN generates poor accuracies. (**a**) Original SAR images after preprocessing; (**b**) adaptive WMM filtering; (**c**) MLP results; (**d**) Final results after post processing.

Figure 4 shows two sample test images from cases where non-adaptive WMM & PCNN generates poor accuracies. The results of adaptive WMM filter and MLP segmentation are presented in the second and third row of Figure 4, respectively. The fourth row presents the final results after post processing.

A not-well-defined massive dark spot and a not-well-defined linear dark spot are displayed in the right and left columns, respectively. Not-well-defined dark spots occur either when a fresh oil spill is present on a bright background or when the background is heterogeneous, resulting in a large number of false alarms after applying the model.

The accuracy of the entire test dataset increased with 1.1%; moreover, standard deviation shows a significant improvement 1.3 (94.65% with a standard deviation of 2.5) in comparison to the same dataset segmented by non-adaptive WMM & PCNN (93.53% with a standard deviation of 3.8).

In the worst case, an accuracy of 87% was produced which is higher than the worst case accuracy presented in Taravat *et al.* (which is 84.88%). The results of the accuracy assessment applied to the different types of anomalies are displayed in Tables 1 and 2.

The approach generates almost similar accuracies on well-defined dark spots in comparison to the accuracies of well-defined dark spots segmented by non-adaptive WMM & PCNN. A significant improvement of 2.46% in accuracy (with the improvement of 0.44 in standard deviation and 2.4% in commission error) has been detected on the not well-defined dark spot dataset segmented by adaptive WMM & MLP in comparison with the same dataset segmented by non-adaptive WMM & PCNN (not well-defined linear dark spots accuracy is 90.36% with a standard deviation of 2.36 and commission error of 10.32%. Not well-defined massive dark spots accuracy is 89.81% with a standard deviation of 2.22 and commission error of 11.70%).

The worst accuracies are 87.00% with 10.3% commission error and 87.5% with 11.4% commission error which are obtained for not well-defined linear dark spots and not well-defined massive dark spots that are 2% with 3.3% commission error and 2.62% with 2.8% commission error higher than the worst accuracies obtained by non-adaptive WMM & PCNN for not well-defined linear dark spots and not well-defined massive dark spots, respectively.

MLP Neural Networks (as a pixel based classification model) are less sensitive to noise and give good performance for spots with weak edges because they utilize the statistical information within or outside the training set and this is the reason of the higher accuracies obtained by adaptive WMM & MLP for not well-defined linear dark spots and not well-defined massive dark spots in compared to the accuracies obtained by non-adaptive WMM & PCNN for not well-defined linear dark spots and not well-defined massive dark spots.

## 4. Conclusions

In the present study a detailed research on the ability of using adaptive WMM & MLP as an improved method of non-adaptive WMM & PCNN model for dark-spot detection in SAR imagery is demonstrated. Adaptive WMM model presented in this study overcomes the non-adaptive WMM filter setting parameters. Furthermore a pixel-based classification model (MLP neural network) has been applied to a test dataset including 60 ENVISAT-ASAR and ERS2-SAR images. The same parameters were used for all the test images.

The whole test dataset accuracy is 94.65%, which is higher than the same dataset segmented by non-adaptive WMM & PCNN (93.53%). The approach generates almost similar accuracy on well-defined dark spots in comparison to the accuracy on well-defined dark spots segmented by non-adaptive WMM & PCNN. Results showed that this approach works better in the situations (not well-defined linear dark spots and not well-defined massive dark spots) where non-adaptive WMM & PCNN generates poor accuracy.

Determination of appropriate characteristics for the training data and number of layers and nodes in the network topology are the main difficulties experienced in the use of many machine learning models, but once the topology and the other parameters are set, it can be used easily and very fast. The proposed approach can be applied well to the other spaceborn SAR (*i.e.*, Sentinel-1) with some parameter adjustment based on the type of data.

## Figures and Tables

**Table 1.** The average values of the accuracies for different types of anomalies.

**DARK Spot Types**	**Min%**	**Max%**	**Mean%**	**StDev**
Well-Defined	95.50	98.00	96.70	0.64
Linear Well-Defined	95.50	97.80	96.50	0.59
Massive Well-Defined	96.00	98.00	96.98	0.62
Not Well-Defined	87.00	94.00	92.55	1.81
Linear Not Well-Defined	87.00	94.00	92.97	2.00
Massive Not Well-Defined	87.50	93.10	92.13	1.58
Linear Dark Spot	87.00	97.80	94.74	2.31
Massive Dark Spot	87.50	98.00	94.55	2.74

**Table 2.** The average values of emission and commission error (In %) achieved by adaptive WMM & MLP.

**Dark Spot Types**	**Min Om**	**Max Om**	**Mean Om**	**StDev Om**	**Min Cm**	**Max Cm**	**Mean Cm**	**StDev Cm**
Well-Defined	2.00	4.50	3.25	0.64	1.10	3.50	2.30	0.62
Linear Well-Defined	2.20	4.50	3.48	0.59	1.10	3.50	2.24	0.62
Massive Well-Defined	2.00	4.00	3.01	0.62	1.40	3.20	2.37	0.64
Not Well-Defined	6.00	13.0	7.44	1.81	6.20	11.4	8.60	1.36
Linear Not Well-Defined	6.00	13.0	7.00	2.00	6.20	10.3	8.22	1.04
Massive Not Well-Defined	6.90	12.5	7.86	1.58	6.50	11.4	8.97	1.58
Linear Dark Spot	2.20	13.0	5.20	2.31	1.10	10.3	5.23	3.17
Massive Dark Spot	2.00	12.5	5.44	2.74	1.40	11.4	5.67	3.57
